# Supplementary material for: Multi‐dataset identification of innovative feature genes and molecular mechanisms in keratoconus
Source: J Cell Mol Med. 2024 Sep 19;28(18):e70079. doi: 10.1111/jcmm.70079 (PMC11412914; doi:10.1111/jcmm.70079)
Supplement: Supplementary file 2 — Material S2. [file JCMM-28-e70079-s002.zip › 7.GSEA analysis/3.DBNDD1/KEGG_P53_SIGNALING_PATHWAY.html]

Details for gene set KEGG\_P53\_SIGNALING\_PATHWAY[GSEA]

|  || Dataset | DBNDD1.group.cls#h\_versus\_l |
| Phenotype | group.cls#h\_versus\_l |
| Upregulated in class | l |
| GeneSet | KEGG\_P53\_SIGNALING\_PATHWAY |
| Enrichment Score (ES) | -0.36190233 |
| Normalized Enrichment Score (NES) | -1.3356959 |
| Nominal p-value | 0.15459883 |
| FDR q-value | 0.8051628 |
| FWER p-Value | 0.945 |
Table: GSEA Results Summary

  

Fig 1: Enrichment plot: KEGG\_P53\_SIGNALING\_PATHWAY      
 Profile of the Running ES Score & Positions of GeneSet Members on the Rank Ordered List

  

| SYMBOL | TITLE | RANK IN GENE LIST | RANK METRIC SCORE | RUNNING ES | CORE ENRICHMENT || 1 | CCNE2 | na | 4812 | 0.186 | -0.1071 | No |
| 2 | TP73 | na | 5519 | 0.164 | -0.0996 | No |
| 3 | RCHY1 | na | 5908 | 0.153 | -0.0847 | No |
| 4 | TP53AIP1 | na | 6088 | 0.148 | -0.0646 | No |
| 5 | APAF1 | na | 8227 | 0.099 | -0.1093 | No |
| 6 | TSC2 | na | 8882 | 0.088 | -0.1132 | No |
| 7 | CASP8 | na | 9432 | 0.079 | -0.1155 | No |
| 8 | CCND1 | na | 9676 | 0.076 | -0.1096 | No |
| 9 | MDM2 | na | 9756 | 0.075 | -0.0992 | No |
| 10 | IGF1 | na | 10555 | 0.064 | -0.1113 | No |
| 11 | SHISA5 | na | 10574 | 0.064 | -0.1010 | No |
| 12 | PTEN | na | 10959 | 0.059 | -0.1021 | No |
| 13 | BID | na | 11063 | 0.057 | -0.0952 | No |
| 14 | ATM | na | 11600 | 0.051 | -0.1020 | No |
| 15 | DDB2 | na | 13284 | 0.036 | -0.1444 | No |
| 16 | CASP3 | na | 13339 | 0.035 | -0.1400 | No |
| 17 | BAX | na | 13486 | 0.034 | -0.1383 | No |
| 18 | TP53 | na | 13962 | 0.030 | -0.1469 | No |
| 19 | CCNG1 | na | 14357 | 0.027 | -0.1535 | No |
| 20 | PPM1D | na | 14388 | 0.027 | -0.1498 | No |
| 21 | TP53I3 | na | 14988 | 0.023 | -0.1631 | No |
| 22 | ATR | na | 15758 | 0.017 | -0.1823 | No |
| 23 | MDM4 | na | 16162 | 0.015 | -0.1914 | No |
| 24 | CD82 | na | 16243 | 0.014 | -0.1913 | No |
| 25 | SESN1 | na | 16426 | 0.013 | -0.1943 | No |
| 26 | CCNE1 | na | 17317 | 0.007 | -0.2188 | No |
| 27 | PERP | na | 17373 | 0.007 | -0.2191 | No |
| 28 | CCND2 | na | 17929 | 0.003 | -0.2346 | No |
| 29 | PIDD1 | na | 19024 | -0.005 | -0.2654 | No |
| 30 | EI24 | na | 19470 | -0.008 | -0.2768 | No |
| 31 | CASP9 | na | 19881 | -0.012 | -0.2866 | No |
| 32 | CYCS | na | 20083 | -0.014 | -0.2900 | No |
| 33 | SFN | na | 21113 | -0.023 | -0.3158 | No |
| 34 | SERPINB5 | na | 21123 | -0.023 | -0.3121 | No |
| 35 | CDK4 | na | 21131 | -0.023 | -0.3084 | No |
| 36 | CDKN2A | na | 21295 | -0.025 | -0.3088 | No |
| 37 | CCNB3 | na | 21737 | -0.029 | -0.3165 | No |
| 38 | CCND3 | na | 22320 | -0.035 | -0.3273 | No |
| 39 | RRM2B | na | 22356 | -0.036 | -0.3222 | No |
| 40 | THBS1 | na | 22482 | -0.037 | -0.3195 | No |
| 41 | SESN2 | na | 22734 | -0.040 | -0.3199 | No |
| 42 | TNFRSF10B | na | 22768 | -0.040 | -0.3140 | No |
| 43 | STEAP3 | na | 23822 | -0.054 | -0.3352 | No |
| 44 | PMAIP1 | na | 24630 | -0.065 | -0.3473 | No |
| 45 | FAS | na | 24662 | -0.066 | -0.3370 | No |
| 46 | ZMAT3 | na | 25517 | -0.079 | -0.3482 | Yes |
| 47 | CDK1 | na | 25994 | -0.087 | -0.3470 | Yes |
| 48 | GADD45G | na | 26045 | -0.088 | -0.3334 | Yes |
| 49 | SIAH1 | na | 26484 | -0.097 | -0.3294 | Yes |
| 50 | CCNB1 | na | 26847 | -0.104 | -0.3221 | Yes |
| 51 | CDK6 | na | 27414 | -0.117 | -0.3185 | Yes |
| 52 | CCNB2 | na | 27443 | -0.118 | -0.2992 | Yes |
| 53 | CHEK2 | na | 27531 | -0.119 | -0.2814 | Yes |
| 54 | RPRM | na | 27608 | -0.121 | -0.2629 | Yes |
| 55 | SESN3 | na | 28222 | -0.136 | -0.2574 | Yes |
| 56 | CDK2 | na | 28335 | -0.139 | -0.2369 | Yes |
| 57 | GADD45A | na | 28540 | -0.145 | -0.2181 | Yes |
| 58 | BBC3 | na | 29642 | -0.177 | -0.2197 | Yes |
| 59 | CCNG2 | na | 29966 | -0.188 | -0.1970 | Yes |
| 60 | GTSE1 | na | 30340 | -0.199 | -0.1738 | Yes |
| 61 | GADD45B | na | 30544 | -0.205 | -0.1447 | Yes |
| 62 | CHEK1 | na | 31495 | -0.240 | -0.1312 | Yes |
| 63 | CDKN1A | na | 31724 | -0.250 | -0.0952 | Yes |
| 64 | IGFBP3 | na | 33020 | -0.330 | -0.0763 | Yes |
| 65 | SERPINE1 | na | 33289 | -0.352 | -0.0239 | Yes |
| 66 | RRM2 | na | 33650 | -0.383 | 0.0311 | Yes |
Table: GSEA details [plain text format]

  

Fig 2: KEGG\_P53\_SIGNALING\_PATHWAY      
 Blue-Pink O' Gram in the Space of the Analyzed GeneSet

  

Fig 3: KEGG\_P53\_SIGNALING\_PATHWAY: Random ES distribution      
 Gene set null distribution of ES for **KEGG\_P53\_SIGNALING\_PATHWAY**
